# Identification of subclinical tuberculosis in household contacts using exposure scores and contact investigations

**DOI:** 10.1186/s12879-020-4800-y

**Published:** 2020-01-31

**Authors:** Gry Klouman Bekken, Christian Ritz, Sumithra Selvam, Nelson Jesuraj, Anneke C. Hesseling, T. Mark Doherty, Harleen M. S. Grewal, Mario Vaz, Synne Jenum

**Affiliations:** 1 Department of Clinical Science, Faculty of Medicine, University of Bergen, Bergen, Norway; 2 Department of Nutrition, Exercise and Sports, University of Copenhagen, Copenhagen, Denmark; 3 Division of Epidemiology, Biostatistics and Population Health, St. John’s Research Institute, Koramangala, Bangalore, 560 034 India; 4 Paediatrics and Neonatology, Trinity hospital, Palakkad, Kerala India; 5 Department of Pediatrics and Child Health, Desmond Tutu TB Center, Stellenbosch University, Cape Town, South Africa; 6 GlaxoSmithKline Vaccines Wavre, Wavre, Belgium; 7 Department of Microbiology, Haukeland University Hospital, N-5021 Bergen, Norway; 8 Division of Health and Humanities, St. John’s Research Institute, Koramangala, Bangalore, 560 034 India; 9 Department of Infectious Diseases, Oslo University Hospital, Pb 4956 Nydalen, 0424 Oslo, Norway

**Keywords:** Pulmonary tuberculosis, Cohort studies, Transmission, Risk assessment, Point scoring systems, Prevention

## Abstract

**Background:**

The goal of tuberculosis elimination put forward in the End TB Strategy prioritizes diagnosis and treatment of incipient and subclinical TB, recently defined by key stakeholders as *“asymptomatic, early pre-clinical disease during which pathology evolves”*. Regarded as indicative of a high risk of TB progression, considerable efforts have been made to identify these cases through exploration of biomarkers. The present study aimed to evaluate simple scoring systems for TB exposure as screening tools for subclinical TB, the only identifiable of the incipient and subclinical disease states, in a contact investigation (CI) setting of low HIV-prevalence.

**Methods:**

Nested within a large prospective study in household contacts (HHCs) of smear positive pulmonary TB cases in South-India conducted 2010–2012, we assessed 1) the association between the Tuberculosis Contact Score (TCS) and the Infectivity Score, with established tools for *Mycobacterium tuberculosis (Mtb)* infection, corrected for established TB risk factors, and 2) the capability of the TB exposure scores to identify subclinical TB defined by *Mtb-*culture positivity in sputum or gastric aspirate (subjects < 5 years) specimen.

**Results:**

Of 525 HHCs, 29 were *Mtb*-culture positive and 96.6% of these asymptomatic. The TCS and the Infectivity Score associated with positive Tuberculin Skin Test and QuantiFeron TB-Gold In-tube assay (QFT) results in multivariate analyses (TCS: OR_TST_ 1.16, 95% CI: 1.01, 1.33; OR_QFT_ 1.33 95% CI: 1.16, 1.51. Infectivity Score: OR_TST_ 1.39, 95% CI: 1.10, 1.76; OR_QFT_ 1.41 95% CI: 1.16, 1.71). The Infectivity Score showed a moderate capability to identify subclinical TB (AUC of 0.61, 95% CI: 0.52, 0.70).

**Conclusions:**

Although our results did not identify an easily applicable screening tool for subclinical TB, the present study indicates that focusing on TB-related symptoms in CI settings may be of limited value for early identification of HHCs with high risk for TB progression.

## Background

Globally, tuberculosis (TB) caused by *Mycobacterium tuberculosis* (*Mtb*) is the largest killer among infectious diseases, causing an estimated 1.2 million deaths in 2018 [1]. Despite declining TB incidence achieved through considerable joint efforts since the Stop TB Initiative [2], the TB epidemic will represent a great challenge for years to come: the estimated number of new TB cases was 10.0 million in 2018 [1]. Traditionally, treatment of cases has been the cornerstone of epidemiological control [2, 3]. In most high-endemic countries, case detection relies on patients seeking health-care because of symptoms, but this strategy leaves 40% of estimated TB cases undiagnosed [4]. In order to reduce the TB prevalence further, The End TB Strategy included in the Sustainable developmental goals, emphasizes early diagnosis of cases [5] including incipient TB defined as *“asymptomatic, early pre-clinical disease during which pathology evolves, such as mycobacterial replication or the inflammatory response. Radiological abnormalities or positive microbiological tests may or may not be present. This state may either evolve and lead to symptomatic clinical TB or regress and remain asymptomatic”* [6]. Meeting this ambitious goal will require systematic screening of contacts and high risk groups for TB disease and subsequent preventive or full-length TB treatment where adequate [5].

Being recently exposed, *Mtb-*infected household contacts (HHCs) have increased risk of TB progression and are therefore defined a target population for active case-finding as part of contact investigations (CIs) [7]. The World Health Organization (WHO) strongly recommends CIs by health staff visiting households following diagnosis of all smear positive pulmonary TB cases. In low-middle income countries (LMICs) with TB incidence ≥100 per 100,000 population, CIs include evaluation for active TB in persons of all ages with symptoms. If active TB is excluded, preventive treatment should be given to children aged < 5 years and persons living with HIV (PLHIV) [8]. Although suggested as a possible strategy to eliminate TB [9–11], giving preventive treatment to all *Mtb*-infected subjects is not practical in LMICs with moderate-high TB incidence as health systems are already overwhelmed. Although the Tuberculin Skin Test (TST) and/or interferon-gamma release assays (IGRAs) give evidence of *Mtb*-infection, these tests have poor predictive value for future TB [12, 13] reducing their relevance in the identification of incipient and subclinical TB [14]. These disease states represent early TB disease with high risk of TB progression and are likely to contribute to continued transmission [6, 14]. Therefore, the World Health Organization (WHO) and other stakeholders are strongly encouraging and facilitating the development of novel tests capable of identifying incipient and subclinical TB [6, 15]. CI represents a reasonable well-established framework for identification of these TB cases which would have a large impact on the TB epidemic [9, 16] subsequently increasing national incentives to adhere to programmatic CIs [8]. Although the scientific community are making progress in identifying host biomarker-based risk signatures for TB progression [17–19], validation and approval according to defined target product profiles [6, 15] will still take time.

Notably, risk factors for incipient/subclinical and active TB can be assumed to be similar [14, 20–22]. The risk of *Mtb-*infection and disease progression is generally accepted to be strongly affected by the degree of exposure [23, 24]. Therefore, a scoring system has been developed to quantify TB-exposure in HHCs when evaluating the performance of the TST and IGRAs in pediatric and adult populations [24–27]. The Tuberculosis Contact Score (TCS) contains multiple subscores (*Infectivity Score, Duration of Contact Score, Type of Exposure Score and Duration of Symptoms Score*) and, as opposed to TST and IGRAs, does not require repeated visits and laboratory facilities. Sputum smear grade alone has also been found to associate with TST [28, 29] and QFT positivity [30, 31]. We therefore hypothesized that TB-exposure scores could serve as screening tools for incipient/subclinical TB. Verification of incipient TB according to the recent consensus definition by Kik et al., is indeed challenging [6]. Inspired by Drain et al. who denotes subclinical TB as a categorical state between incipient and clinical TB where cases can be identified by microbiological or radiological evidence [14], we considered laboratory confirmation a more robust evidence of ongoing *Mtb-*replication than chest X-ray changes. We therefore defined subclinical TB by growth of *Mtb* in respiratory (or gastric aspirate for children aged > 5 years) specimens from recently exposed HHCs and argue that we hereby identify the subjects with the highest bacterial burden suggestive of risk of transmission and progression to overt disease. Patient-reported symptoms are highly variable in previous Asian studies even in definite TB cases [32], and were therefore not included in our definition.

Thus, in the context of a prospective study of HHCs of adult pulmonary TB index cases in South India, our primary aim was to evaluate the potential of the TB exposure scores TCS and the Infectivity Score (a TCS subscore), as screening tools for subclinical TB. We first assessed the association between the TB exposure scores for identifying *Mtb-*infection (TST and Quantiferon) and subclinical TB while correcting for established TB risk factors. We then evaluated the capability of the TB exposure scores to identify individual contacts and/or families with ≥1 subclinical TB case.

## Methods

### Study population

The present study was a cross-sectional study nested within a large prospective household contact (HHC) study conducted in Palamaner Taluk, Andhra Pradesh, India (3.200°N, 72.7500°E, altitude 683 m) in the period 2010–2012. The bacillus Calmette-Guérin vaccine (BCG) coverage in the area was > 90% in all populations < 2 years except for the muslim minority (81.4%) [33]. The HIV prevalence in tested pregnant women in the area was 1–2% [34]. Index cases were recruited through the RNTCP (Revised National TB Control Program) when diagnosed with smear positive pulmonary TB (PTB). Both index cases and household contacts were enrolled in the HHC study: eligible PTB cases were smear positive subjects aged > 18 years. Eligible contacts were persons living ≥75% of the time in the same household as the index case and sharing the same kitchen [35]. Contacts with previous PTB or already on TB treatment/prophylaxis were excluded. Written informed consent was given by all adults. Children aged 7–18 years gave their assents followed by parental consent whereas parental consent alone was given for children aged < 7 years.

### Tuberculosis contact investigation

According to the study protocol, all participating contacts were offered a comprehensive evaluation at baseline. This included an interview on socioeconomic conditions, medical history, TB-symptoms and clinical examinations including weight, TST, the QuantiFeron TB-Gold In-tube assay (QFT), two sputa (gastric aspirates for children ≤5 years) harvested on two consecutive days, for smear and culture, and chest X-rays (anterior view for all, lateral view added on selected children < 5 years). First, a blood sample was drawn for the QFT that was interpreted according to the manufacturer’s instructions (a positive test was defined as ≥0.35 IU/ml). Then trained staff performed a TST by injecting 2 TU Purified Protein Derivate (PPD, SPAN Diagnostics Ltd., Surat, India) intra-dermally on the volar part of the left arm. The following induration was read after 48-96 h (~ 80% evaluated within 72 h, the remaining within 96 h) and defined positive if ≥10 mm. The TST was repeated after 1–4 weeks in HHCs with an induration < 5 mm (*n* = 54), and the baseline TST result defined as the larger of the two tests. The chest X-rays were interpreted as either “normal”, “abnormal, not TB” or “abnormal TB”, first by a medical officer in the field, later by a radiologist whose interpretation was preferred if discrepancy. Sputum/gastric samples were evaluated by smear microscopy for acid-fast bacilli (AFB) and cultured on both liquid (BACTEC MGIT 960™ [Becton and Dickinson, USA]) and solid (Lowenstein-Jensen) media. Identification of *Mtb* was done using the GenoType MTBC test kit (HAIN kit). All HHC participants were offered HIV-testing and pre-test counselling at baseline.

### TB exposure scores: the tuberculosis contact score and the infectivity score

The Tuberculosis Contact Score (TCS) was based on previously published scores when assessing children [24] and adults [26], and modified to include HHCs of all ages. *Relationship score* was excluded as data were judged unreliable (Table 1).

**Table 1:** The Tuberculosis (TB) Contact Score. Maximum score per subject = 18

Tuberculosis Contact Score (TCS)	Pre-assigned weight
Infectivity of the index case^1^	
No known TB contact	0
Unknown sputum smear status	1
Sputum acid-fast^2^ negative	2
Sputum acid-fast^2^ 1+	3
Sputum acid-fast^2^ 2+	4
Sputum acid-fast^2^ 3+	5
Sputum acid-fast^2^ 4+	6
Type of exposure to index case^1^	
No known/unknown exposure	0
Index case lives and sleep in different house	1
Index case lives and sleep in same house	2
Index case lives and sleep in same room	3
Index case lives and sleep in same bed	4
Duration (total hours) of contact per average day with index case^1^	
No known/unknown duration of contact	0
0–3 h	1
4–7 h	2
8–11 h	3
≥ 12 h	4
Duration of TB symptoms in index case^1^	
No symptoms or unknown duration of symptoms	0
< 3 weeks	1
4–7 weeks	2
8–11 weeks	3
≥ 12 weeks	4

^1^Index case: Adult with confirmed pulmonary tuberculosis. ^2^Direct fluorescent microscopy with Auramin staining

The TCS was based on interview (three questions) and sputum evaluation of the index cases assuming the gradient of exposure to be a composite function of: 1) the infectivity of the index case represented by the sputum smear grade, graded 0–6 (*Infectivity Score*), 2) closeness to the index case during sleep, graded 0–4 (*Type of Exposure Score*), 3) the time (hours per day) spent with the index case, graded 0–4 (*Duration of Contact Score*), and 4) duration of the index case’s symptoms before first visit to the doctor, graded 0–4 (*Duration of Symptoms Score*). The TCS is a sum of all subscores (max score 18); the higher the score, the greater the exposure and risk of *Mtb-*infection and disease.

### Categorization and definition of household contacts (HHCs) according to *Mtb-*infection status and subclinical TB

For analysis, HHCs were categorized according to their *Mtb-*infection status at baseline defined by 1) TST and QFT results (both tests required: TST and QFT negative; TST or QFT positive; TST and QFT positive) or 2) subclinical TB defined by positive *Mtb*-culture in sputum or gastric aspirate (subjects < 5 years) specimen. Although being asymptomatic is the key in the suggested definition for subclinical TB, broad reports from similar CI settings in Asia suggest that 40–79% of active TB cases do not report symptoms [32] highlighting the disease continuum and difficulty in drawing a strict line between subclinical and active TB. After all, finding all these cases must be the goal of CIs. In accordance with the suggested subclinical TB definition [14] and the study aim of finding a screening tool applicable in the field, categorization of subclinical TB was done irrespective of chest X-ray results.

### Statistical analysis

Categorical variables were reported as percentages. Continuous variables were summarized by mean and standard deviation or median and interquartile range, as appropriate. TST (mm) and QFT (IU/ml) results were analyzed both as continuous variables and as categorical variables dichotomized around their cut-offs (TST ≥10 mm) and (QFT ≥0.35 IU/ml). Distribution of clinical characteristics between HHCs categorized according to *Mtb*-infection status was assessed with Chi-square test and one-way analysis of variance (ANOVA) (Tukey “Honest Significant Difference” method for post-hoc comparisons), where appropriate. Associations between the independent variables TCS and established risk factors for TB (age, gender, BCG-scar, diabetes, smoking, indoor pollution, crowding) and the dependent variables 1) TST and QFT results, and 2) subclinical TB, were evaluated by univariate logistic regression, and multivariate logistic regression adjusted for previously listed risk factors for TB. Since assessment of body weight is different in subjects aged ≥15 years (Body Mass Index, BMI) and < 15 years (Body Mass Index Z-score, BMIZ), BMI and BMIZ were only entered in the models evaluating this variable, which were then stratified for age. All other listed TB risk factors were entered in the BMI/BMIZ models. Similar models were fitted to the Infectivity Score. Unadjusted, predictive capabilities of TCS, the Infectivity Score, TST, and QFT for the individual contacts and/or families with ≥1 contact *Mtb* culture positive at baseline, were assessed using receiver operating characteristic (ROC) curves and area under curve (AUC). A significance level of *p* < 0.05 was used. IBM SPSS Software, version 25 and R Core Team, 2018.

## Results

### Characterization of the study population

A total of 525 household contacts (HHCs) were recruited from 176 index cases in the study area accounting for 94.3% of all eligible households. Index cases reported a median of 20 days (IQR: [15, 30] days) from onset of symptoms to first doctor visit, and a median of 6 days (IQR: [3, 20] days) from doctor visit to diagnosis. At inclusion, 93 index cases (57%) had started treatment. In these subjects, the median time from diagnosis to initiation of treatment was 2 days (IQR: [2, 4] days), and a median of 2 days of treatment was given at the time of CI (IQR: [1, 2] days). Baseline investigations were completed in 490 (93%) HHCs, in a median of 4 days (IQR: [3, 5] days) in the 436 (83%) HHCs with only one TST, and a median of 17.5 days (IQR: [15.0, 19.25]) in the 54 (10%) HHC with repeated TST. Adequate specimen for Mtb culture were harvested in 493 (93.9%) HHCs. Of these, 488 (99%) (and all 38 children < 5 years) had two samples harvested on two consecutive days.

Males accounted for 40.5% of the study cohort, and 63% of the study cohort were aged > 14 years. BCG scar was recorded in 221 (51.5%). All the 312 (60.4%) HHCs that agreed to HIV-testing were HIV negative. Houses were small with a mean of 4.25 (St.dev 2.04) subjects per room. Indoor pollution can be assumed high as 75% used wood and agricultural residue for fuel. In addition, 118 (24.8%) were/had been smokers for ≥6 months. The distribution of gender, age, TB risk factors and clinical parameters within the *Mtb*-infection categories are given in Table 2.

**Table 2:** Distribution of age, gender and risk factors for tuberculosis (TB) in 525 household contacts of 176 adults with confirmed pulmonary TB according to infection categories at baseline Contact Investigation

	Missing	Total with available data *N* = 525	TST−/QFT-^1^ *N* = 167	TST+ or QFT + ^1^ *N* = 167	TST+/QFT + ^1^ *N* = 113	Subclinical TB^2^ at baseline *N* = 29
	n (%)	n (%)	n (%)	n (%)	n (%)	n (%)
*Interview*						
Gender (male)	49 (9.3)	193 (40.5)	79 (47.3)	61 (36.5)	39 (34.5)	14 (48.3)
Age (years)^3^	50 (9.5)					
< 5		38 (8.0)	22 (13.2)	8 (4.8)	6 (5.4)	2 (6.9)
5–14		138 (29.1)	41 (24.6)	47 (28.1)	38 (33.9)	12 (41.4)
> 14		299 (62.9)	104 (62.3)	112 (67.1)	68 (60.7)	15 (51.7)
Individual risk factors						
Diabetes mellitus	49 (9.3)	9 (1.9)	2 (1.2)	5 (3.0)	1 (0.9)	1 (3.4)
Smoking	49 (9.3)	118 (24.8)	35 (21.0)	48 (28.7)	27 (23.9)	8 (27.6)
Tuberculosis Contact Score						
Mean (St.dev)		10.9 (2.3)	10.4 (2.3)	11.1 (2.3)	11.6 (2.0)	11.2 (1.8)
Symptoms^4^		13 (2.7)	5 (3.0)	4 (2.4)	3 (2.7)	1 (3.4)
Environmental risk factors						
Crowding^5^ (mean, St.dev)	55 (10.5)	4.25 (2.04)	4.41 (2.25)	4.14 (1.98)	4.18 (1.89)	3.43 (1.53)
In-door pollution^6^	55 (10.5)					
LPG		60 (11.4)	14 (9.8)	21 (14.2)	17 (16.3)	2 (7.4)
Kerosene		15 (2.9)	3 (2.1)	4 (2.7)	5 (4.8)	1 (3.7)
Wood + agri residue		329 (75.2)	126 (88.1)	123 (83.1)	82 (78.8)	24 (88.9)
*Examination and tests*						
Tuberculin Skin Test (TST)	49 (9.3)					
mm (median, range)		8.0 (34)	6.0 (9)	7.0 (34)	14.0 (23)	7.0 (12)
≥ 10 mm		152 (31.9)	0	30 (18.0)	113 (100)	9 (31.0)
Quantiferon (QFT)	51 (9.7)					
IU/mL (median, range)		0.56 (19.8)	0.03 (3.2)	1.44 (10.7)	4.58 (9.6)	1.36 (10.1)
≥ 0.35 IU/mL		266 (56.1)	0	137 (82.0)	113 (100)	16 (59.3)
*Mtb* specimen for culture	32 (6.1)					
2 of 2 samples harvested		471 (98.9)	162 (97.9)	167 (100)	113 (100)	29 (100)
1 of 2 samples harvested		5 (1.1)	5 (3.0)	0	0	0
Smear +		7 (1.5)	2 (1.2)	2 (1.2)	2 (1.8)	1 (3.4)
Chest X-ray	49 (9.3)					
Abnormal, not TB		13 (2.7)	4 (2.4)	1 (0.6)	7 (6.2)	1 (3.4)
Abnormal, TB		15 (3.2)	3 (1.8)	7 (4.2)	3 (2.7)	2 (6.9)
BCG scar present	96 (18.3)					
Yes		221 (51.5)	85 (55.6)	76 (50.7)	46 (46.0)	14 (53.8)
BMI and BMIZ^7^						
BMI when aged ≥15 years. Mean (st.dev)	29 (8.8)	19.6 (3.19)	19.30 (3.03)	20.15 (3.72)	19.25 (2.61)	18.69 (2.13)
BMIZ when aged< 15 years. Mean (st.dev)	9 of 187	−0.14 (1.70)	− 0.48 (1.85)	0.19 (1.33)	−0.14 (1.91)	− 0.15 (1.71)

^1^Tuberculin Skin Test (TST) was dichotomized around the cutoff 10 mm; ≥10 mm corresponding to TST+ and < 10 mm corresponding to TST-. Quantiferon (QFT) was dichotomized around the cutoff 0.35 IU/mL; ≥0.35 IU/mL corresponding to QFT+ and < 0.35 IU/mL corresponding to QFT-. Analysis limited to subjects with both test results. ^2^Subjects with growth of *Mycobacterium tuberculosis* (Mtb) in liquid culture of respiratory specimen. ^3^WHO based age categories.^4^ ≥ 1 of the following: cough > 2 wks, fever/night sweats, loss of weight/appetite, haemoptysis, chestpain. ^5^Number of household members divided on rooms in the house. ^6^By type of fuel: LPG, Kerosene, Wood + agricultural residue. ^7^Body Mass Index (*BMI*), Body Mass Index Z-score (*BMIZ*)

Among the 29 subclinical TB cases, one (5.5%) had symptoms, one had a positive smear and “abnormal TB” chest X-rays, one had “abnormal TB” and one “abnormal, not TB” chest X-ray.

### Association between TB exposure scores and *Mtb*-infection in HHCs

Although the association between TCS and TST/QFT is established in other cohorts [24–27], we first investigated the strength of this association in the present Indian cohort corrected for established risk factors for TB. The TCS and the Infectivity Score was associated with positive TST and QFT both in univariate and in multivariate analysis (Table 3).

**Table 3:** Associations between Tuberculosis Contact Score, the Infectivity Score and other risk factors for tuberculosis (TB) and the dependant variables TST^1^ and QFT^1^ in 525 household contacts of 161 adults with pulmonary TB

	TST- vs TST + ^1^			QFT- vs QFT + ^1^		
	univariate	multivariate^2^	multivariate^3^	univariate	multivariate^2^	multivariate^3^
	OR (95%CI)	OR (95%CI)	OR (95%CI)	OR (95%CI)	OR (95%CI)	OR (95%CI)
Tuberculosis Contact Score	**1.15 (1.05, 1.26)**	**1.16 (1.01, 1.33)**		**1.22 (1.12, 1.32)**	**1.33 (1.16, 1.51)**	
InfectivityScore	**1.28 (1.11, 1.48)**		**1.39 (1.10, 1.76)**	**1.25 (1.11, 1.42)**		**1.41 (1.16, 1.71)**
Gender	1.44 (0.97, 2.13)	1.35 (0.85, 2.15)	1.37 (0.87, 2.18)	1.38 (0.95, 1.99)	1.38 (0.88, 2.16)	1.47 (0.94, 2.28)
Age (years)^4^						
< 5	0.44 (0.19, 1.02)	0.71 (0.27, 1.87)	0.72 (0.28, 1.88)	**0.46 (0.23, 0.91)**	0.87 (0.36, 2.11)	0.81 (0.34, 1.92)
5–14	1.07 (0.70, 1.62)	1.05 (0.61, 1.82)	1.05 (0.61, 1.82)	1.20 (0.79, 1.81)	1.27 (0.74, 2.17)	1.32 (0.77, 2.24)
> 14	ref	ref	ref	ref	ref	ref
BCG scar present	0.93 (0.62, 1.38)	1.04 (0.65, 1.65)	1.02 (0.64, 1.63)	**0.66 (0.45, 0.97)**	0.64 (0.40, 1.01)	0.63 (0.40, 1,00)
Individual risk factors						
Diabetes mellitus	0.26 (0.03, 2.09)	0.22 (0.03, 1.83)	0.22 (0.03, 1.84)	2.80 (0.58, 13.61)	3.41 (0.62, 18.69)	3.26 (0.60, 17.79)
Smoking	1.21 (0.78, 1.86)	1.64 (0.93, 2.89)	1.67 (0.94, 2.96)	1.18 (0.77, 1,79)	1.15 (0.65, 2.04)	1.15 (0.65, 2.03)
≥ 1 individual risk factors	1.19 (0.77, 1.82)			1.21 (0.80, 1.84)		
BMI^5a^	0.98 (0.90, 1.05)	0.96 (0.86, 1.06)	0.96 (0.88, 1.06)	1.03 (0.96, 1.11)	0.97 (0.88, 1.06)	0.99 (0.90, 1.08)
BMIZ^5b^	1.03 (0.86, 1.24)	na	na	1.16 (0.97, 1.38)	na	na
Environmental risk factors						
Crowding^6^	1.01 (0.91, 1.11)	1.09 (0.97, 1.22)	1.09 (0.97, 1.22)	0.92 (0.84, 1.01)	0.99 (0.88, 1.11)	0.97 (0.87, 1.08)
In-door pollution^7^						
LPG	**1.78 (1.01, 3.15)**	**2.05 (1.07, 3.93)**	**1.96 (1.02, 3.76)**	1.48 (0.81, 2.68)	1.28 (0.65, 2.54)	1.25 (0.63, 2.47)
Kerosene	1.89 (0.62, 5.76)	2.17 (0.67, 7.08)	2.34 (0.72, 7.59)	1.80 (0.55, 5.96)	1.15 (0.33, 4.09)	1.37 (0.39, 4.83)
Wood + agri residue	ref	ref	ref	ref	ref	ref

^1^Tuberculin Skin Test (*TST*) was dichotomized around the cutoff 10 mm; ≥10 mm corresponding to TST+ and < 10 mm corresponding to TST-. Quantiferon (*QFT*) was dichotomized around the cutoff 0.35 IU/mL; ≥0.35 IU/mL corresponding to QFT+ and < 0.35 IU/mL corresponding to QFT- (Subjects with indeterminate QFT is excluded from analysis). ^2^Analysis including the TCS; ^3^Analysis including the Infectivity Score. ^4^WHO based age categories. ^5^Since assessments of body weight is different in ^5a^subjects aged ≥15 years (Body Mass Index, *BMI*) and ^5b^ < 15 years (Body Mass Index Z-score, *BMIZ*), BMI and BMIZ were only entered in the models evaluating this variable, which were then done stratified for age. ^6^Number of household members divided on rooms in the house. ^7^Grading based on type of fuel, (Wood + agricultural residue) = 3, Kerosene = 2, LPG = 1

ORs in bold are significantly different from 1 (*p*<0.05)

In the multivariate models, the likelihood for a positive TST increased by 16% (95% CI: 1–33%) per unit increase in the TCS and by 39% (95% CI: 10–76%) per unit increase in the Infectivity Score, whereas the likelihood for a positive QFT increased 33% (95% CI: 16–51%) per unit TCS and 41% (95% CI: 16–71%) per unit increase of the Infectivity Score. Surprisingly, LPG fuel, indicative of higher SES and less indoor pollution, was significantly associated with a positive TST in multivariate analysis (TCS model: OR 2.05, 95% CI: 1.07, 3.93. Infectivity Score model: OR 1.96, 95% CI: 1.02, 3.76). Notably, the association was not present when analyses were limited to HHCs ≥15 years regardless of BMI being included in analyses. No other known TB risk factors associated with TST or QFT result.

### Association between TB exposure scores and subclinical TB in HHCs

There was no association between the TCS or the Infectivity Score and subclinical TB (Table 4).

**Table 4:** Associations between Tuberculosis Contact Score, the Infectivity Score and other risk factors for tuberculosis (TB) and the dependent variables TST^1^ and QFT^1^ in 525 household contacts of 161 adults with pulmonary TB

	Subclinical TB vs other HHCs		
	univariate	multivariate^2^	multivariate^3^
	OR (95% CI)	OR (95% CI)	OR (95% CI)
Tuberculosis Contact Score	1.07 (0.90, 1.27)	0.98 (0.76, 1.27)	
InfectivityScore	1.31 (0.96, 1.80)		1.35 (0.87, 2.12)
Gender	0.71 (0.34, 1.52)	0.78 (0.32, 1.85)	0.75 (0.32, 1.81)
Age (years)^4^			
< 5	1.05 (0.23, 4.80)	2.14 (0.37, 12.3)	2.39 (0.42, 13.7)
5–14	1.80 (0.82, 3.96)	2.63 (0.87, 7.96)	2.71 (0.88, 8.33)
> 14	ref	ref	ref
BCG scar present	1.11 (0.50, 2.45)	1.01 (0.41, 2.50)	0.99 (0.40, 2.44)
Individual risk factors			
Diabetes mellitus	1.96 (0.24, 16.2)	1.53 (0.17, 14.0)	1.73 (0.19, 16.2)
Smoking	1.17 (0.50, 2.71)	1.61 (0.49, 5.27)	1.63 (0.49, 5.38)
≥ 1 individual risk factors	1.11 (0.48, 2.58)		
BMI^5^	0.89 (0.73, 1.09)	0.92 (0.73, 1.16)	0.92 (0.73, 1.16)
BMIZ^5^	1.00 (0.73, 1.38)	0.87 (0.61, 1.24)	0.82 (0.56, 1.19)
Environmental risk factors			
Crowding^6^	**0.79 (0.62, 0.99)**	**0.72 (0.54, 0.95)**	**0.71 (0.53, 0.95)**
In-door pollution^7^			
LPG	0.53 (0.12, 2.31)	0.45 (0.10, 2.08)	0.38 (0.08, 1.78)
Kerosene	1.15 (0.14, 9.22)	0	0
Wood + agri residue	ref	ref	ref

^1^Tuberculin Skin Test (*TST*) was dichotomized around the cutoff 10 mm; ≥10 mm corresponding to TST+ and < 10 mm corresponding to TST-. Quantiferon (*QFT*) was dichotomized around the cutoff 0.35 IU/mL; ≥0.35 IU/mL corresponding to QFT+ and < 0.35 IU/mL corresponding to QFT- (Subjects with indeterminate QFT is excluded from analysis). ^2^Analysis including the TCS. ^3^Analysis including the Infectivity Score. ^4^WHO based age categories. ^5^Since assessments of body weight is different in ^5a^subjects aged ≥15 years (Body Mass Index, BMI) and ^5b^ < 15 years (Body Mass Index Z-score, *BMIZ*), BMI and BMIZ were only entered in the models evaluating this variable, which were then done stratified for age. ^6^Number of household members divided on rooms in the house

ORs in bold are significantly different from 1 (*p*<0.05)

Of TB risk factors, only crowding was associated with subclinical TB (TCS multivariate model: OR 0.72, 95%CI: 0.54, 0.95. Infectivity Score multivariate model: OR 0.71, 95% CI: 0.53, 0.95). This negative association was unexpected as crowding normally increases TB risk [36]. Notably, crowding was not significant in the BMI model, suggesting interaction between BMI and crowding. To explore this, the interaction BMI/crowding was entered in the model resulting in no significant association for crowding alone.

### The capacity of TB exposure scores to identify subclinical TB

**Fig. 1:**
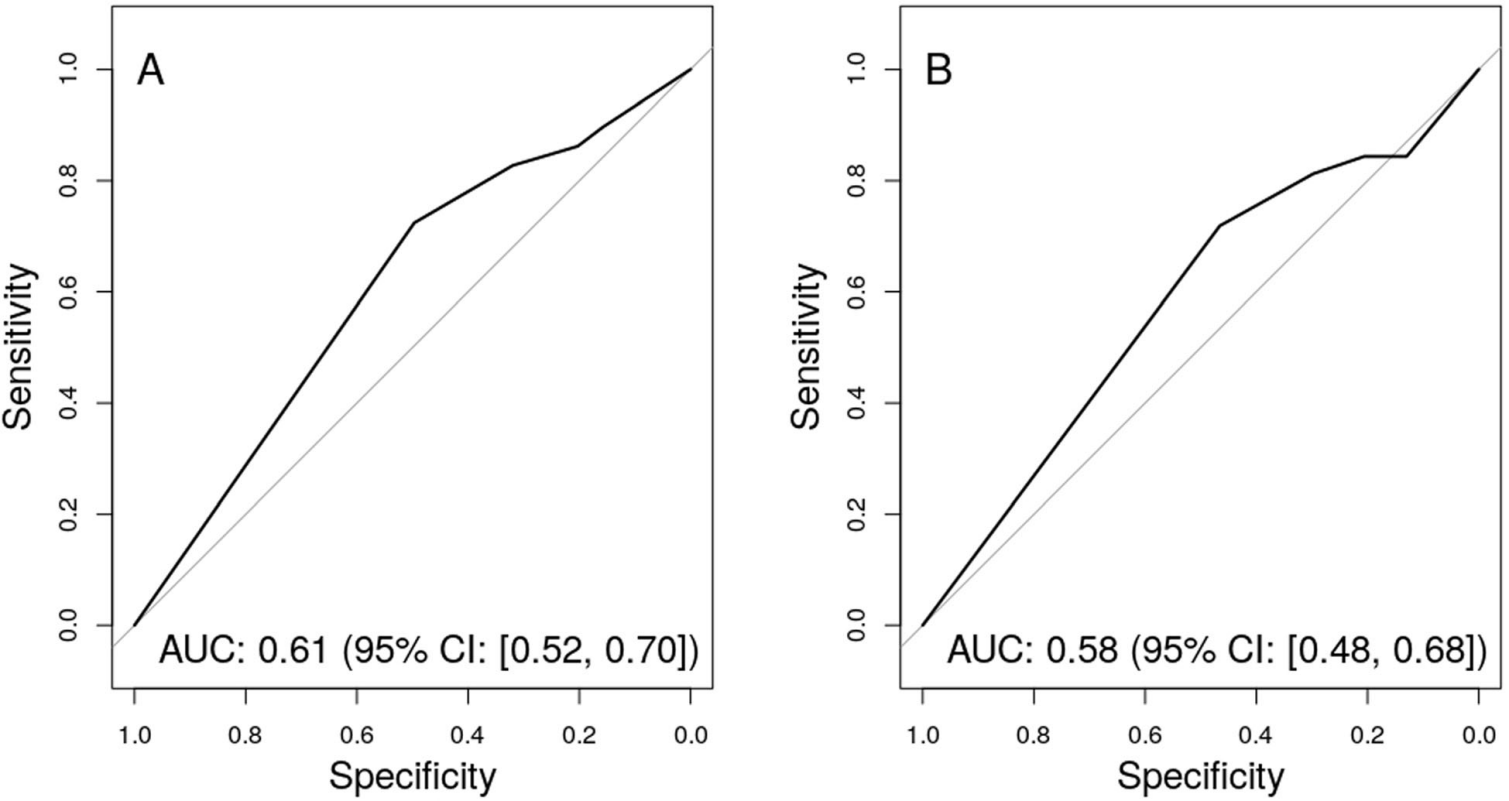
The capability of the Infectivity Score to identify individual household contacts (HHCs) with growth of *Mtb* in sputum cultures (A) and families with ≥1 HHCs (B)

Finally, we assessed the capability of the TB exposure scores to identify individual contacts and/or families with ≥1 contact with subclinical TB. For comparison, the same analyses were performed for TST and QFT at baseline CI. As our aim was to evaluate the potential of the TB exposure scores as robust screening tools, no adjustments were made. The TCS and the established immunological tools, TST and QFT, all had AUC values close to 0.5 indicating no capability to identify individual HHCs and/or families with ≥1 HHC with *Mtb*-positive cultures (data not shown). Interestingly, the simpler Infectivity Score showed moderate capability to identify individual contacts (AUC of 0.61, 95% CI: 0.52, 0.70) but marginal capability to identify families with ≥1 contact with growth of *Mtb*-positive sputum cultures (AUC of 0.58, 95% CI: 0.48, 0.68) (Fig. 1).

## Discussion

To our knowledge, the present study is the first to assess the Tuberculosis Contact Score (TCS) and the Infectivity Score as screening tools for subclinical TB in a CI setting. Currently, no tools are available for this purpose. Even with promising host biomarker-based risk signatures for TB progression [17–19], validation in various populations as well as translation to a point-of-care test format, will take time [6, 15]. Therefore, the present study was motivated by our hope that simple scoring systems, previously proven to reflect TB exposure, a well-established risk factor for TB, could fill some of this gap. Being independent of laboratory and X-ray facilities, the TCS or the Infectivity Score can easily be applied as a screening tool resulting in more targeted CIs either by sorting out low-risk families/subjects where no CI could be justified, or identify high-risk families/subjects warranting referral for further TB investigations. Regrettably, the TCS could not reliably identify individual subjects or families with subclinical TB. The Infectivity Score performed better with a significant capacity to identify subjects and families with subclinical TB with an AUC of 0.61 and 0.58 respectively. This is however, not good enough for a screening test [37] to identify low-risk (“rule-out”) or high-risk (“rule-in”) families/subjects, underlining the need for continued search for host biomarkers for this screening purpose. Nevertheless, the present study adds an important aspect for CIs. Notably, the strong recommendations for CI for active case finding in all households of sputum positive TB index patients [8, 38] focus on symptomatic HHCs. The present study, one of very few studies from India where sputum samples for both smear and culture were collected from all HHCs regardless of signs and symptoms of disease, provides clear evidence that the majority of HHCs with replicating *Mtb* in respiratory specimen were asymptomatic. The discrepancy between symptoms, X-ray findings and *Mtb-*culture results is remarkable, and illustrates the challenge of early identification of cases based on patient-reported symptoms. Among the 525 HHCs, 29 met our definition of subclinical TB (5.5%), but only 1 of the *Mtb-*culture positive participants had symptoms qualifying for a co-prevalent case (0.2%). Of 525 HHCs, 13 (2.7%) reported symptoms, but only one of the symptomatic HHCs (8.3%) had *Mtb*-positive cultures. Therefore, in a regular CI setting where microbiological sampling is, at best, done only in symptomatic subjects [5], 28 of 29 (96.6%) subclinical cases would remain undetected. The frequency of *Mtb*-culture positivity of 5.5% HHCs in the present study, is remarkably high compared to the reported 0.23% of *Mtb*-culture positivity in symptomatic Indian patients (passive case-finding) [39, 40], but comparable to another Indian Study (4.3%) [41]. We do though, acknowledge that our definition of subclinical TB that relied on *Mtb-*culture results only, and not on chest X-ray findings as suggested by Drain et al. [14], might have underestimated the number of subclinical TB cases. Interestingly, only 2 (6.9%) had X-ray changes suggestive of TB and none of these had symptoms. The same picture was observed for HHCs within the other *Mtb*-infection categories with X-ray changes suggestive of TB: Of 3 TST−/QFT- subjects none had symptoms, of 6 TST+ or QFT+ one had symptoms, and of 3 TST+/QFT+ none had symptoms. Therefore, we argue that our *Mtb-*culture based definition of subclinical TB is the most objective and robust tool for identification of subjects with high-risk for progressive disease and transmission [42–45]. We acknowledge though, that the relevance of *Mtb-*replication and transient excretion in the early phase of *Mtb*-infection reported in children [46], that probably also occurs in adults, could be questioned, as successful containment and spontaneous recovery can be expected in an unknown proportion of subjects [14].

The window of HHC enrollment following TB index case identification could influence the number of HHCs with ongoing *Mtb*-replication verified by positive cultures. The present study succeeded in rapid inclusion of HHCs illustrated by 42.6% of TB index cases not yet on treatment at inclusion. The delay to diagnosis for the index cases was higher in our study (mean 20 days) than in other Indian studies (mean 6–16 days) [47–49], but lower than in LMICs other than Sub-Saharan Africa (median 27 days) [50]. Furthermore, the majority of included HHCs had baseline investigations efficiently completed within a median of 4 days (83%), and all completed within 40 days.

Of further relevance is the vulnerability for *Mtb-*infection and subclinical TB in our population for which the presence of TB risk factors in the study population could be indicative: Despite a high reported BCG-coverage [33], only 51.1% of all HHCs in our study had a BCG scar compared to > 80% in a semi-urban population in Delhi [41]. Interestingly, scar rates of only 47.5% were reported in newborns with low birthweight [51]. A prospective cohort study conducted (2006–2008) in the same study area reported a birthweight of ≤2500 g in 29% of 4382 neonates [52] which taken together suggests an explanation for the low BCG scar rate in the present study.

Surprisingly, and despite the fact that we for the first time confirmed a clear association between the TB exposure scores and TST/QFT in an Indian HHC population, neither the TB exposure scores or other well-established TB risk factors had a clear association with subclinical TB in multivariate analysis. There are multiple possible explanations: Although high TB exposure outside the household is the most evident, this seems unlikely given the Indian TB incidence about 180 per 100,000 in the study period [4]. It may be possible that the moderate size of our study cohort means that it is not entirely comparable with findings from large epidemiological studies [21, 22, 36]. Furthermore, it is possible that the considerable crowding (mean of 4.25 persons per room) have affected negatively the performance of the TCS by causing less differentiated exposure of the HHCs to the index case. Crowding might also decrease the association observed in large-scale studies between smoking and TB disease due to passive smoking. The size of the households in the present study (median of 5) was similar to another HHC study in rural Indian [53]. Semi-urban and urban Indian families tend to be smaller [41, 54], but congested living is common in both rural and urban areas [41] with 96.8% of families in the present study living in 1–2 rooms. Interestingly, multivariate analyses revealed a possible interaction between BMI and crowding that could confound the results. Notably, children aged < 15 could not be included in multivariate analyses assessing BMI as BMI is an invalid measurement in this age group. For HHCs aged ≥15 years, HHCs with and without subclinical TB had a mean BMI of 18.7 (St.dev 2.13) and 19.6 (St.dev 3.25) respectively. This was considerably lower than the BMI in two large Peruvian HHC Study cohorts (mean BMI 25.2 and 25.6). Interestingly, the dose-response log-linear relationship between BMI and TB incidence reported in a meta-analysis, was less certain at BMI < 18.5 and > 30 [21].

The strengths of the present study are inclusion of HHCs of all ages, detailed sociodemographic data, a thorough work-up of most contacts with TST, QFT, sputum or gastric aspirate samples for both smear and cultures, combined with data on the TCS and Infectivity Score for all HHCs. A major weakness is the lack of 18–24 months follow-up data in accordance with current consensus definition for incipient TB [6]. Another limitation is due to the 18% missing data in the study. The missing data may be the reason that some associations may not have been detected. Moreover, as the missingness pattern is at least partly attributable to missing not at random mechanisms (e.g., different types of non-responders) there is a risk of bias in the reported associations.

## Conclusions

Although our results were disappointing with regard to our hope of identifying an easily applicable screening tool for subclinical TB, the present study provides relevant information to CIs as framework for identification and early treatment of subclinical TB required to achieve the ambitious goal of the End TB Strategy [5]. Findings in our cohort indicate that focusing on TB-related symptoms is of little value in identifying HHCs with replicating *Mtb* in respiratory specimens, as this strategy would have left 96.6% of HHCs with *Mtb*-positive cultures unidentified. Although other studies support more targeted identification of subjects with high TB risk based on other TB risk assessment-based framework [21, 22, 36], it is questionable whether exploring and validating eventual new TB risk scores offers a viable alternative to host biomarker-based screening tools for subclinical TB, which are strongly needed to reach the ambitious goal of TB elimination.

## Data Availability

The datasets generated and/or analysed during the current study are not publicly available due to ongoing work on immune readouts in the same cohort but are available from the corresponding author on reasonable request.

## Abbreviations

ANOVA: Analysis of variance
AUC: Area under curve
BCG: Bacillus calmette-guérin vaccine
BMI: Body mass index
BMIZ: Body mass index Z-score
CI: Contact investigation
HHC: Household contact
HIV: Human immunodeficiency virus
IGRA: Interferon-gamma release assay
IQR: Interquartile range
LMICs: Low-middle income countries
*Mtb*: *Mycobacterium tuberculosis*
OR: Odds ratio
PLHIV: People living with human immunodeficiency virus
PPD: Purified protein derivate
PTB: Pulmonary tuberculosis
QFT: Quantiferon TB-gold In-tube assay
RNTCP: Revised national TB control program
ROC: Receiver operating characteristic
TB: Tuberculosis
TCS: Tuberculosis contact score
TST: Tuberculin skin test
WHO: World health organization
